# Bio- Fortification of *Angelica gigas* Nakai Nano-Powder Using Bio-Polymer by Hot Melt Extrusion to Enhance the Bioaccessibility and Functionality of Nutraceutical Compounds

**DOI:** 10.3390/ph13010003

**Published:** 2019-12-25

**Authors:** Md Obyedul Kalam Azad, Wie Soo Kang, Jung Dae Lim, Cheol Ho Park

**Affiliations:** 1 Department of Bio-Health Technology, College of Biomedical Science, Kangwon National University, Chuncheon 24341, Korea; azadokalam@gmail.com (M.O.K.A.); kangwiso@kangwon.ac.kr (W.S.K.); 2 Department of Herbal Medicine Resource, Kangwon National University, Samcheok 25949, Korea; ijdae@kangwon.ac.kr

**Keywords:** *Angelica gigas* nakai, biopolymer, hot melt extrusion, nanocomposite, phenolic compound, antioxidant capacity, solubility, bioaccessibility

## Abstract

*Angelica gigas* Nakai (AGN) is a popular traditional herbal medicine which has been used to alleviate various human diseases in Korea since ancient times. However, the low bioaccessibility of the nutraceutical compounds of AGN results in a poor water solubility, thereby limiting bioavailability. In this regard, a ternary AGN–biopolymer–plasticizer composite (AGNC) was developed to enhance the bioaccessibility of nutraceutical compounds from extrudate AGN formulations manufactured by hot melt extrusion (HME). The AGNC was prepared with extrudate AGN (EAGN) using different hydroxypropyl methylcellulose (HPMC) biopolymers (5% *w*/*w*) viz.: hypromellose phthalate (HP), hypromellose (AN), and hypromellose (CN) along with acetic acid (AA) (0.1 M, 20% *w*/*v*) as a plasticizer. The non-extrudate fresh AGN (FAGN) powder was used as a control. The physicochemical properties of the extrudate formulations and control were characterized by differential scanning calorimetry (DSC) and Fourier-transform infrared spectroscopy (FTIR). DSC analysis showed a lower enthalpy (ΔH) (12.22 J/g) and lower glass transition temperature (*T*_g_) (41 °C) in HP-AA-EAGN compared to the control. FTIR confirmed the physical crosslinking between AGN and biopolymer in the extrudate composite and demonstrated that some functional groups formed viz., -OH and -CH_2_. The obtained result also shows that the particle size was reduced by 341 nm, and solubility was increased by 65.5% in HP-AA-EAGN compared to the control (1499 nm, 29.4%, respectively). The bioaccessibility of the total phenolic content and the total flavonoids—including decursin (D) and decursinol angelate (DA)—were significantly higher in HP-AA-EAGN compared to the control. The 2,2-diphenyl-1 picryl hydrazyl (DPPH) free radical scavenging capacity and ferric reducing antioxidant power assay (FRAP) indicated that the HP-AA-EAGN formulation preserves a greater antioxidant profile than the other formulations. Finally, it is summarized that the addition of acidified HP biopolymer increased the bioaccessibility, functionality, and improved the physicochemical properties of nutraceutical compounds in the extrudate AGN formulation.

## 1. Introduction

*Angelica gigas* Nakai (AGN) is a traditional herbal medicine which has also been used as a functional food in Korea since ancient times. It is well recognized that AGN possesses several nutraceutical compounds which have strong antioxidant capacities [1]. AGN also has numerous pharmacological properties such as the ability to cure menopausal syndromes, anemia, and abdominal pain, in addition to the ability to inhibit breast cancer and amenorrhea [2,3]. However, the poor water solubility of the AGN compounds makes it less functional [4]. The main reason for this poor water solubility is the lipophilicity of its bioactive compounds, its strong intermolecular covalent bonds, and its large molecular weight [5].

**Figure 1:**
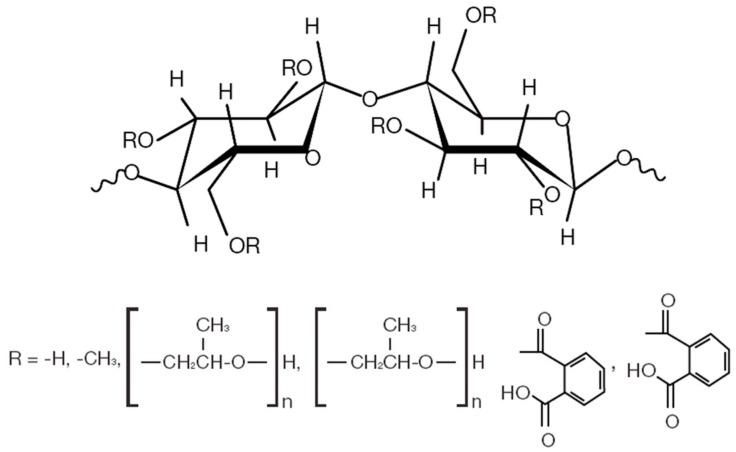
Chemical structure of hydroxypropyl methylcellulose (HPMC) and their substitute groups.

Recently, biopolymers—mainly cellulose and semi-synthetic cellulose—have been garnering increasing consideration in the food industry as a means of improving the functionality of nutraceutical compounds. Cellulose polymers are an entirely safe food additive which are recommended by the European Food Safety Association (EFSA) [6]. Cellulose ethers and esters are two main groups of cellulose derivatives with different physicochemical and mechanical properties (Figure 1). Among the different biopolymers, hydroxypropyl methylcellulose (HPMC) is a hydrophilic biocompatible hydrocolloid with excellent film-forming properties, mainly used in the food industry as a stabilizer, an emulsifier, and a protective colloid [7]. It has hydration and gel-forming capabilities which enhance solubility, reduce particle size, control the delivery system in the intestines, and control the pH-dependent dissolution behavior of the active ingredients [8,9]. The encapsulating and binding capacities of the HPMC polymer mostly depend on the presence in their chemical structure of an ether or ester group and their molecular weight, as well as their degree of viscosity. Cellulose ether is hydrophilic and has the ability to form hydrogel after exposure to aqueous media. Likewise, cellulose esters are water-insoluble polymers with an excellent film-forming capacity, which are widely used in enteric-coating drug delivery systems [10]. The chemical nature and efficiency of the HPMC are dependent on the amount of the hydropropoxyl and methoxyl content and the substitution of the ester or ether group. In general, the chemical attributes of different types of biopolymers are almost the same, but the application is different. For instance, a low viscosity polymer (<10 mPa.s) is used as a film coating in an aqueous solvent, a moderate viscosity polymer (40–100 mPa.s) is used in enteric coating and a high viscosity polymer (>4000 mPa.s) is used in sustained release and as a thickening agent. Hydrophilic cellulose ether and ester derivatives are commonly used for improving the film forming and plasticity of nutraceutical compounds [11].

**Figure 2:**
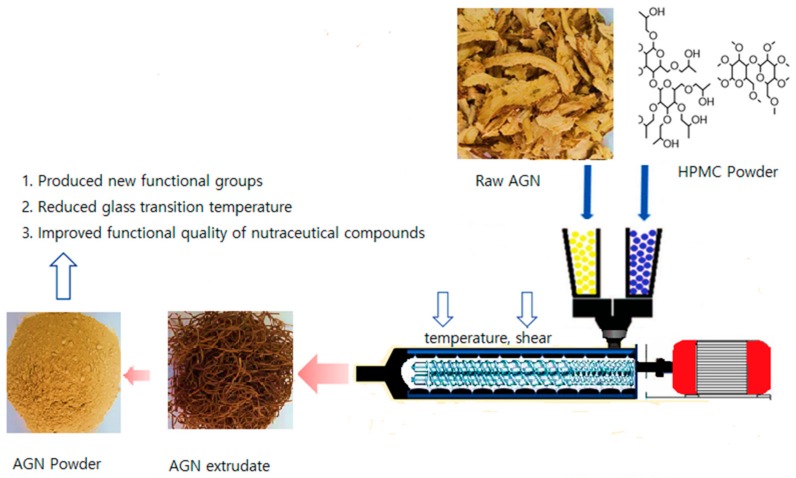
Schematic illustration of a hot melt extrusion process.

The enhanced aqueous solubility of the active compound is achieved by the amorphization of its crystalline compounds, using hydrophilic polymers through hot melt extrusion by steric hindrance and non-covalent bonding within the polymeric chain [12]. The aqueous solubility of bioactive compounds plays a vital role in determining their permeability and absorption rate in the body. Many approaches have been investigated to improve the solubility of the bioactive compound; among them, particle size reduction is one of the most effective ways to increase solubility [13]. The extrusion process is traditionally employed via ram extrusion and screw (single or twin screw) extrusion. Twin screw extrusion generates high shear stress with a higher degree of mixing capacity than the single screw extruder. Hot melt extrusion (HME) equipped with twin screw is widely used in the pharmaceutical and food industries, to develop solid composites with a nanosized particle of the active compounds [14] (Figure 2). The HME extrusion process is generally carried out above the gelling temperature of the polymer components, to achieve the optimal miscibility of the active compounds and polymer [15]. HME technology facilitates the generation of new compounds and produces oligosaccharide from polysaccharide in extrudate materials [16]. In general, the high temperature of the HME softens the polymer and favors the diffusion of active compounds such that they are dispersed into the polymer matrix. The degradation of excipients during extrusion can be alleviated by the application of plasticizers such as stearic acid, acetic acid, citric acid, triethyl citrate, and salicylic acid [17,18]. The processing ability in the melt extrusion process can be enhanced by adding a plasticizer, which lowers the melt viscosity of the extrudate. Hydrophilic plasticizers have a significantly positive effect on the increase in dissolution rate. During the HME process, biopolymers are depolymerized, and polymer chains break down due to thermal and high shear stress [14]. During the HME process, the active compounds are supposed to be dispersed into a polymeric matrix where active compounds are present as dissolved particles or as a solid solution [19].

In our previous study, the applicability and efficiency of HPMC biopolymer in the formulation of a soybean nanocomposite were evaluated [20]. This study showed that the HPMC biopolymer enhanced the bioaccessibility of secondary compounds from soybeans, and also prepared a nanosized particle. However, to the best of our knowledge, there is no research published on the preparation of AGN-biopolymer solid formulation in order to improve their functionality. In this regard, an *A. gigas* Nakai composite was developed using the HPMC biopolymer with a plasticizer (acetic acid), to enhance the bioaccessibility of the nutraceutical compounds. It is hypothesized that a polymer-plasticizer-based AGN composite would potentially improve the functionality of the nutraceutical compounds from *A. gigas* Nakai.

## 2. Materials and Methods

### 2.1. Preparation of AGN Powder

The dried root of the AGN was purchased from the Chuncheon local market (Chuncheon, Korea). The root was then blended using an electric blender to make AGN powder. The AGN powder was milled into a fine powder by a pin crusher (JIC-P10-2; Myungsung Machine, Seoul, Korea). The powder was passed through 500 µm sieves to achieve a uniform particle size. The AGN powder was stored in a desiccator for further use.

### 2.2. Preparation of AGN–Biopolymer–Plasticizer Composite Formulation and HME Configuration

Three types of food grade hydroxypropyl methylcellulose (HPMC) biopolymers were donated by the Lotte Food Co. Ltd., Seoul, Korea. The HPMC was graded according to its functional groups and physicochemical characteristics (Table 1). The AGN–biopolymer–plasticizer composite (AGNC) was prepared with 5% of HPMC (*w*/*w*) along with 0.1 M acetic acid (AA) (20% *w*/*v*) by the hot melt extrusion (HME) described in Table 2. The HME (STS-25HS twin-screw, Hankook E.M. Ltd., Pyoung Taek, Korea) was equipped with a round-shaped die (1 mm) at a feeding rate of 40 g/min, rpm 220 with high shear stress (a pressure range of 100–150 bar). The temperature profile of the HME barrel from the feeding zone to die was 80/100/120/80 °C. The HME temperatures were set above the gelling temperature of the HMPC, to facilitate the miscibility of the AGN powder and biopolymer. The AGNC solid extrudate was dried in an oven at 50 °C then ground for further analysis.

### 2.3. Analysis of the Physicochemical Properties of the AGNC Formulation

#### 2.3.1. Particle Size Analysis

The particle size of the AGNC and the control were calculated using a light-scattering spectrophotometer (ELS-Z1000; Otsuka Electronics, Tokyo, Japan). A total of 0.3 g of powder of the formulations was suspended in 30 mL of distilled water. The mixture was centrifuged at 5000 rpm for 10 min to separate the supernatant to analyze the particle size. The particle size and distribution for each formulation were measured 70 times with three replications to ascertain an average particle size and particle distribution range.

#### 2.3.2. Water Absorption Index, Water Solubility, and Swelling Power Analysis

One gram of the AGNC and control powders was suspended in 50 mL of distilled water; the mixture was stirred for 1 h at room temperature and then centrifuged at 5000 rpm for 10 min. The supernatant was decanted into an evaporating dish of known weight. The moisture content of the sample was measured by the moisture meter, and between 2% and 3% of the moisture was used to measure the water-related parameters. The water absorption index (WAI), water solubility (WS), and swelling power (SP) were calculated by the following formulas described by Piao et al. [21].

$$\mathrm{Water}\;\mathrm{Absorption}\;\mathrm{Index}\;(\mathrm{WAI})\;=\;\frac{\mathrm{wet}\;\mathrm{sediment}\;\mathrm{weight}}{\mathrm{dry}\;\mathrm{sample}\;\mathrm{weight}}\;$$

$$\mathrm{Water}\;\mathrm{Solubility}\;(\mathrm{WS})\;=\;\frac{\mathrm{dry}\;\mathrm{supernatant}\;\mathrm{weight}}{\mathrm{dry}\;\mathrm{sample}\;\mathrm{weight}}\times \;100$$

$$\mathrm{Swelling}\;\mathrm{Power}\;(\mathrm{SP})\;=\;\frac{\mathrm{wet}\;\mathrm{sediment}\;\mathrm{weight}}{\mathrm{dry}\;\mathrm{sample}\;\mathrm{weight}\;\times \;\left(\;1-\frac{\mathrm{WS}\;\left(\%\right)}{100}\;\right)}\;$$

#### 2.3.3. Differential Scanning Calorimetry (DSC) Analysis

The glass transition temperatures of the AGNC and control formulations were obtained by DSC analysis (DSC Q2000, TA Instruments, New Castle, DE, USA). Approximately 2.0 ± 0.1 mg of samples was put on aluminum crucibles under a nitrogen atmosphere, at a flow of 50 mL min^−1^ in the temperature range of 20 °C to 250 °C, with a heating rate of 10 °C min^−1^. Indium (melting point 156.6 °C) was used as the standard for equipment calibration. Data were analyzed using the software Universal Analysis 2000 (TA Instruments, New Castle, DE, USA).

#### 2.3.4. Fourier-Transform Infrared Spectroscopy (FTIR) Analysis

An FTIR analysis of the AGNC formulations was performed by a Perkin–Elmer Model 1600 apparatus (Norwalk, CT, USA). Ten milligrams of each AGNC sample were homogeneously mixed with KBr on the attenuated total reflectance (ATR) plate to prepare translucent disks in the range of 4000 to 400 cm^−1^. For each spectrum, 100 scans were co-added at a spectral resolution of ±4 cm^−1^. The spectrometer was continuously purged with dry nitrogen. The frequencies for all sharp bands were accurate to within 0.001 cm^−1^. Each sample was scanned under the same conditions with three different pellets. The absorption intensity of the peaks was calculated using the base-line method. The ATR plate was carefully cleaned by scrubbing it with 70% isopropyl alcohol twice, after which it was dried with soft tissue before the new sample was analyzed.

### 2.4. Extraction of Nutraceutical Compounds from AGNC Formulation

One gram of the AGNC and control powders was added to 100 mL of 80% ethanol. The samples were sonicated in an electric sonicator for 60 min at 35 °C. The extracts were filtered (4 µm) through Advantech 5B filter paper (Tokyo Roshi Kaisha Ltd., Saitama, Japan) and dried using a vacuum rotatory evaporator (EYLA N-1000, Tokyo, Japan) in a 40 °C water bath to obtain the crude extract. The crude extract was freeze-dried to obtain a moisture content of <2–3%. The dried crude extract was diluted using distilled water to prepare a 1000 mg/L stock solution and kept at −4 °C for further analysis.

#### 2.4.1. Determination of Total Phenolic Content (TP)

Total phenolic content (TP) was determined according to the Folin–Ciocalteu assay [22]. In brief, a sample aliquot of 1 mL of stock solution was added to a test tube containing 0.2 mL of phenol reagent (1 N). The volume was increased by adding 1.8 mL of deionized water, and the solution was vortexed and left for 3 min for a reaction. Furthermore, 0.4 mL of Na_2_CO_3_ (10% in water, *v*/*v*) was added, and the final volume (4 mL) was adjusted by adding 0.6 mL of deionized water. The absorbance was measured at 725 nm by a spectrophotometer after incubation for 1 h at room temperature. The TP was calculated from a calibration curve using gallic acid as a standard and expressed as mg/100 g of gallic acid equivalent on a dry mass basis (dmb).

#### 2.4.2. Determination of Total Flavonoid Content

The total flavonoid content (TF) was determined according to Ghimeray et al. [23] with slight modifications. In brief, a 0.5 mL aliquot of the sample (1 mg/mL) was mixed with 0.1 mL of 10% aluminum nitrate and 0.1 mL of potassium acetate (1 M). To this mixture, 3.3 mL of distilled water was added to make the total volume of 4 mL. The mixture was vortexed and incubated for 40 min. The total flavonoids were measured using a spectrophotometer (UV-1800 240 V, Shimadzu Corporation, Kyoto, Japan) at 415 nm. The TF was expressed as mg/100 g coumarin equivalents on a dry mass basis (dmb).

#### 2.4.3. Analysis of Decursin and Decursinol Angelate

Decursin (D) and decursinol angelate (DA) were analyzed using high-performance liquid chromatography (HPLC). An HPLC system (CBM-20A, Shimadzu) with two gradient pump systems (LC-20AT, Shimadzu), a C18 column (Kinetex, 100 × 4.6 mm, 2.6 micron, Phenomenex), an auto-sample injector (SIL-20A, Shimadzu), a UV-detector (SPD-10A, Shimadzu) and a column oven (35 °C, CTO-20A, Shimadzu) were used for this analysis. Solvent A was 0.4% formic acid in water, and solvent B was acetonitrile. A gradient elution was used (0–15 min, 33%–45% B; 15–30 min, 45%–55% B; 30–40 min, 55%–80% B; 40–45 min, 33%–80% B). The flow rate was 1.0 mL/min, the injection volume was 10 μL, and the detection wavelength was 329 nm. D and DA at concentrations of 10, 20, 40, 60, and 80 μg/mL were prepared as standards.

#### 2.4.4. Antioxidant Capacity Analysis

The antioxidant activity was determined based on the scavenging activity of the stable 2,2-diphenyl-1 picryl hydrazyl (DPPH) free radical according to methods described by Braca et al. [24], with slight modifications. One milliliter of the extract was added to 3 mL of DPPH. The mixture was shaken vigorously and left to stand at room temperature in the dark for 30 min. The absorbance was measured at 517 nm using a spectrophotometer (UV-1800 240 V, Shimadzu Corporation, Kyoto, Japan). The percent inhibition activities of the AGNC sample were calculated against a blank sample using the following equation: inhibition (%) = (blank sample–extract sample/blank sample) × 100. 

The reduced power of the AGNC and control samples was estimated according to the ferric reducing antioxidant power (FRAP) assay, as described by Pulido et al. [25]. In brief, 1 mL of stock solution (1 mg/mL) was mixed with 1 mL of 0.2 M phosphate buffer, maintaining a pH of 6.6. The mixture was then incubated at 50 °C for 20 min. After incubation, 1 mL of trichloro-acetic acid (TCA) was added to the solution and centrifuged at 3000 rpm for 10 min. The collected supernatant was diluted with distilled water at a 1:1 ratio. Finally, 0.25 mL of 0.1% ferric chloride was added, and the absorbance was measured at 700 nm by a spectrophotometer.

### 2.5. Statistical Analysis

All data were expressed as means ± the SD of triplicate measurements. The obtained results were compared among the different formulations of AGNC to observe significant differences at the level of 5%. The paired *t*-test between mean values of the formulation was analyzed by MINITAB version 17.0 (Minitab Inc., State College, PA, USA). The figures have been drawn using the Origin Pro 6.1 (36 Washington St, Suite 221, USA).

## 3. Results and Discussion

### 3.1. Particle Size and Solubility Analysis

**Figure 3:**
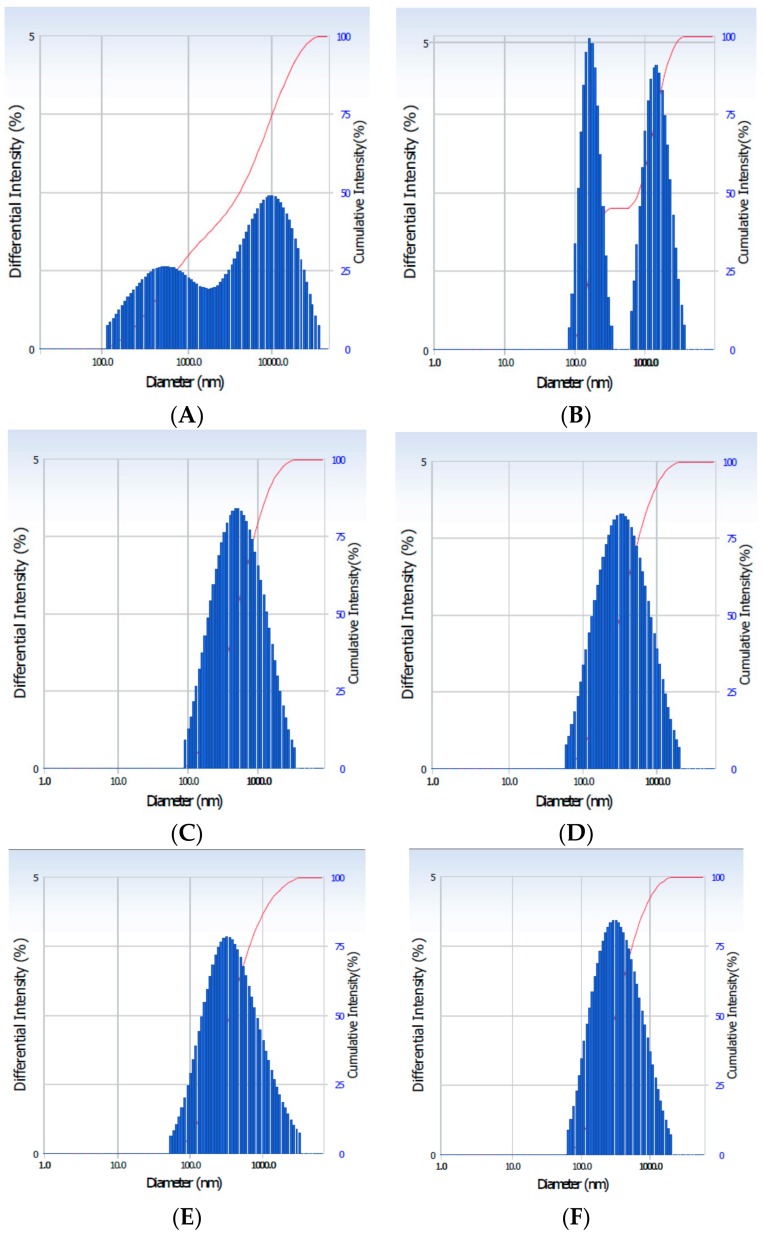
Particle size distribution of the AGNC formulation. (**A**): FAGN; (**B**): EAGN; (**C**): AA-EAGN; (**D**): HP-AA-EAGN; (**E**): CN-AA-EAGN; (**F**): AN-AA-EAGN. HP/CN/AN-AA-EAGN: HPMC (5% *w*/*w*) mediated AA-EAGN.

Particle size reduction is a process of reducing large solid unit masses into small unit masses, coarse particles or fine particles. Normally, size reduction is achieved by different methods, however, a mechanical process using hot melt extrusion is the most commonly used means of forming solid extrudate composites in pharmaceutical industries. The particle size of the AGNC formulations is shown in Table 3, and particle distribution is shown in Figure 3. It is demonstrated that a less nanosized particle (341 nm) was attained in the HP-AA-EAGN (HPMC-mediated acetic acid extrudate AGN) formulation, whereas particle size in the FAGN (fresh non-extrudate AGN) and EAGN (extrudate AGN) formations was 1499 nm and 478 nm, respectively. Particle size was reduced by three times in the EAGN and by five times in the HP-AA-EAGN compared to the FAGN. It is also observed that acidifying biopolymers enhanced the nanonization of the AGNC materials during the HME process.

The high energy of HME breaks down the particles into nanoparticles via a top-down technique [26] and can induce the change to an increased amorphous fraction [27,28]. It is reported that HME improved the solubility of the poorly water-soluble bio-active compound. The HME process helps to convert active crystalline compounds into an amorphous state and dissolves the active ingredient in a polymer matrix through the formation of a solid solution [29]. A decrease in particle size to the few micron ranges and down to the nanosize range can increase the extent and rate of solubility of compounds.

The solubility of an active compound is a major limiting factor for its applicability. Therefore, the solubility enhancement of active compounds is an essential task in the pharmaceutical and food industries, which leads to an improved bioavailability. The water solubility index indicates the total degradation undergone by starch molecules. The increased solubility of extrudate products is attributed to the dispersal of amylose and amylopectin molecules following gelatinization. 

Table 4 shows the water solubility (WS), water absorption index (WAI), and swelling power (WP) of the AGNC formulation. It is indicated that WS increased (65.21%) in HP-AA-EAGN formulations compared to EAGN (42.54%), and FAGN (29.69%). On the other hand, WAI and SP were significantly reduced in HP-AA-EAGN formulations compared to the control, which is technically supposed to be the case.

The solubility enhancement of the poorly water-soluble compounds can be induced by changes to their molecular structure caused by mechanical forces and the preparation of nanocomposites with biopolymers. It is well established that HME is the most suitable process to form nanosized particles [29,30]. The particle size reduction and solubility enhancement directly relate to the increase in contact surface area between the HPMC polymer and bioactive molecules. According to the modified Noyes–Whitney Equation (1) [31], particle size has a direct effect on the solubility and dissolution rate:

(1) $$\frac{\mathrm{dC}}{\mathrm{dt}}=K_{0}A\;(\mathrm{Cs}\;-\;C)$$

where K_0_ is the overall solute transfer coefficient, A is the total contact surface area between solvent and solute, Cs is the saturation solubility of the solid solute and C is the concentration of the solid solute in the bulk solution. In the same way, the dissolution of active compound particles into the molten polymer matrix during HME can be described using the Noyes–Whitney Equation (2)

(2) $$\frac{\mathrm{dm}}{\mathrm{dt}}=\frac{\mathrm{DS}}{\mathrm{Vh}}\;(\mathrm{Cs}\;-\;C)$$

where $\;\frac{\mathrm{dm}}{\mathrm{dt}}=\;\mathrm{solute}\;\mathrm{dissolution}\;\mathrm{rate}\;(\mathrm{kg}/s$); m = mass of the dissolved material (kg); t = time (s); D = diffusion coefficient (m/s); S = surface area of the solute particle (m^2^); C = particle surface concentration (moles/L); h = thickness of the diffusion layer on the solid active compound particle and V = volume of the dissolution medium. Cs = saturation solubility of the solid solute, C = concentration of the solid solute in the bulk solution.

According to the above Equations (1) and (2), a more intimate contact between the particle surface and the aqueous solvent enhances the dissolution rate. During the HME process, the particle surface area increases with a concomitant increase in dissolution rate into the polymer via the diffusion process. It is stated that HPMC improves solubility due to its ability to provide hydrogen bonding donor sites, which prevents migration of the active molecules within the polymer matrix [32,33]. The efficacy of the HPMC to enhance the solubility of the poorly soluble compound is primarily due to its ability to prevent recrystallization of the molecules in the aqueous media [34]. The reduction of WAI and SP due to polymer mixes may possibly be a result of the dextrinization effect during extrusion, resulting in the decomposition of starch.

Also, an acidic solution (H^+^) increases the concentration gradient and enhances the dissolution rate through ionization [33,35]. It is also shown that the HPMC has the greatest stabilizing capacity in a low pH environment. Amorphous nanoparticles exhibit very high saturation solubility compared to the crystalline form [36]. The HME tends to make more channels to enhance the permeability and penetration of water into the core of the matrix of the material [21]. The solubility of amorphous substances is higher than the thermodynamically stable crystalline forms, because their internal bonding forces are weak. To improve the solubility, the HPMC polymer has been used because it readily generates amorphous forms and may be able to retain the amorphous nature of the compound [37]. 

### 3.2. Thermal Analysis of the AGNC Formulation by DSC and FT–IR

Thermal analysis is a technique by which a physical property of a substance is measured as a function of temperature, while the substance is subjected to a controlled temperature program. A complete thermal analysis measures transition temperatures and energies, dimensional changes, and viscoelastic properties. 

The analysis of the thermal characteristics of the melt extrudate active compound and polymers matrix is an essential aspect of solid formulation, enabling knowledge of the status of dissolution, and predicting stability and bioavailability. Thermal analysis shows the behavior of the solid extrudate materials as a function of temperature and time. It provides information about particle morphology and crystallization, as well as solid transformations during heating [14]. The glass transition temperature (Tg) is an important property of an amorphous material. At this temperature, the material behavior changes from a glassy state to a more rubbery state and the mobility of the polymer chain is increased [38].

**Figure 4:**
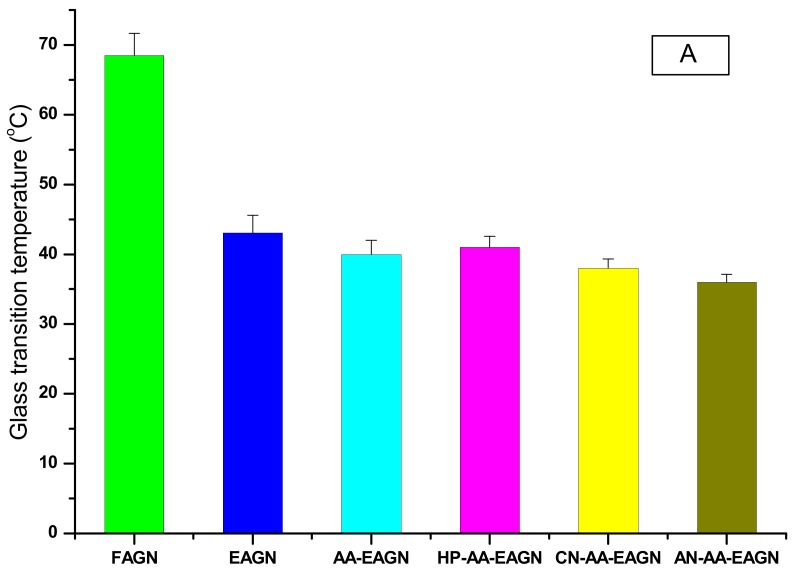

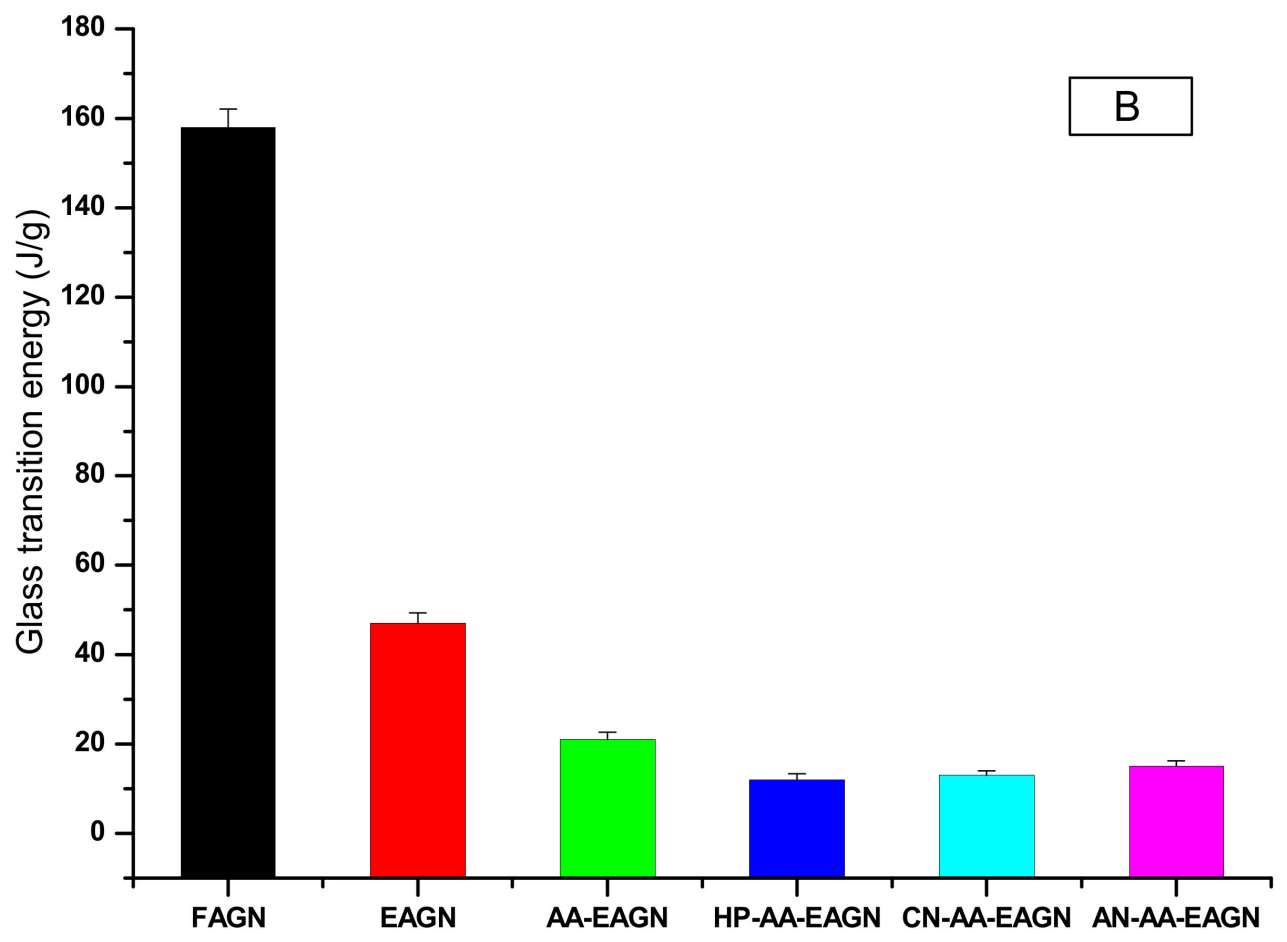
Glass transition temperature (**A**) and glass transition energy (**B**) of the AGNC formulation.

In the DSC analysis, the glass transition temperature (Tg) and glass transition energy (ΔH) are determined (Figure 4). The results of this study indicate that HP-AA-EAGN has a lower Tg (41 °C) and ΔH (12 J/g) compared to FAGN (68.5 °C; 147 J/g, respectively), whereas the Tg and ΔH of EAGN and AA-EAGN were observed at 43 °C, 47 J/g and 40 °C, 21 J/g, respectively. It is reported that amorphous materials have a lower Tg compared to crystalline materials [39]. The ΔH and Tg of the extrudate EAGN appeared as very weak transitions, as more crystalline materials act as physical crosslinks that restrain the mobility of the amorphous regions [40]. It has been previously reported that the Tg of the extrudate formulation can be reduced to 40 °C by adding HPMC polymer [41]. The addition of a plasticizer in the melt extrusion process lowers the Tg and melt viscosity of the extrudate materials. Plasticizers typically work by embedding themselves between polymer chains and minimizing secondary interactions, so that the polymer chain segments are relatively free to move. Therefore, the chain segments maintain their mobility up to a lower temperature than normal. After further cooling, the mobility of the plasticizer freezes at the glass transition temperature. Moreover, the plasticizer decreases the viscosity and increases the free volume, thus significantly lowering the glass transition temperature [42].

In our study, acetic acid (0.1 M) constituted an efficient plasticizer (among citric acid and tartaric acid, data not shown), which reduced the Tg twelve times compared to the control. However, Bruce et al. [43] observed that citric acid has been proven to be an efficient solid-state plasticizer to reduce Tg, improve processability, and increase the release rate of the EUDRAGIT S 100 solid formulation. Citric acid monohydrate has been shown to provide a superior performance compared to anhydrous acid, as a plasticizer [44]. The compatibility and efficiency of acid as a plasticizer depends on the amount of carbon present in the acid molecules and the characteristics of active compounds present in the food materials.

**Figure 5:**
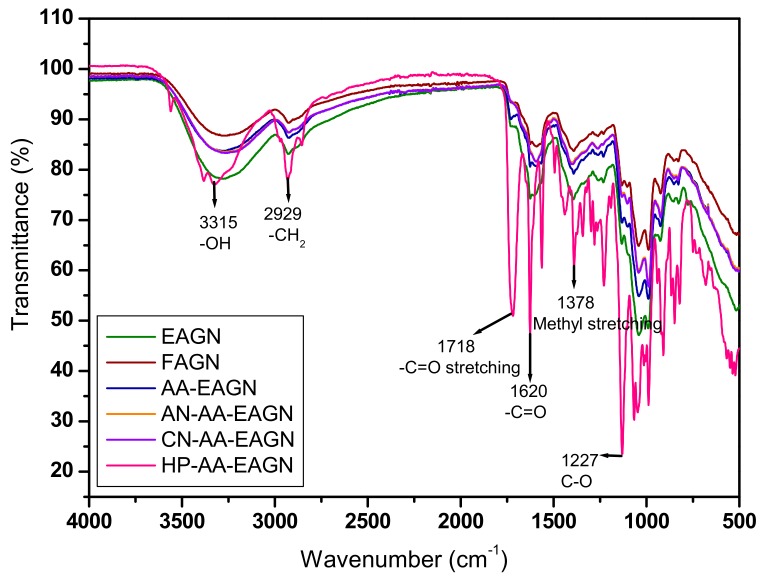
Fourier-transform infrared spectroscopy (FTIR) spectra of AGNC formulations.

Fourier-transform infrared spectroscopy (FTIR) monitors the vibrations of the functional groups that characterize the molecular structure and observes the progress of the shifting and stretching of functional groups. FTIR spectroscopy was used to confirm the physical crosslinking between the AGN and HPMC biopolymers and showed new functional groups produced in the AGNC formulation. The spectra are presented in (Figure 5), which shows that the splitting peak is in the range 1700−3500 cm^−1^ in polymer-mediated extrudate AGNC; however, no splitting peak was observed in FAGN in the same range.

The changes in the FTIR spectra of the AGNC formulations are mainly related to the intensity and position of some bands corresponding to the formation of new functional groups, suggesting an interaction of secondary metabolites with the polymeric matrix [45]. In the HP-mediated extrudate AGNC, there is a new peak in the wavelength of 3315 and 2929 cm^−1^, which corresponds to O–H and CH_2_ stretching. The stretching of alkynes, benzene, and its derivatives occurred near 3500–4000 cm^−1^ in the HP-polymer-mediated formulation. The other prominent peaks in the range 1700–1200 cm^−1^ for biopolymer-extrudate samples possess the characteristics of methylene and methyl bending. The peak region between 3500 and 3000 cm^−1^ is related to C–H and OH compounds (SP^2^), which are attributed to the nature of the organic compounds. Peak regions at <2000 cm^−1^ represent the carbonyl group compounds and the =C bonds in the aromatic rings and aromatic CH bonds on substituted rings [46]. The peaks in the region <1500 cm^−1^ are related to carbon–oxygen bonds (CO) in ethers, esters, and carboxylic acids and are indicative of a wide variety of metabolites, such as tannins, flavonoids, and anthraquinones [47]. FTIR analysis suggested the formation of hydrogen bonding between the secondary compounds of AGN and the hydroxyl group of the HPMC. This likely contributed to the formation of an amorphous solid dispersion [48]. Because of the strong interaction between the AGN compound and the HPMC polymer, it is possible to lower the process temperature and increase the chemical stability of the mixing [49].

### 3.3. Analysis of Total Phenolic Compound and Total Flavonoid Content from AGNC Formulation

**Figure 6:**
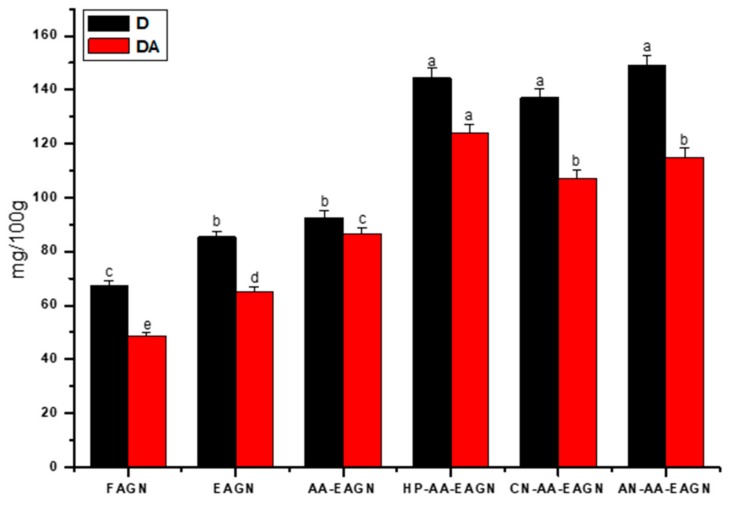
The content of decursin (D) and decursinol angelate (DA) of the AGNC formulation. Values marked by different letters in each column are significantly different in the *t*-test (*p* < 0.05).

**Figure 7:**
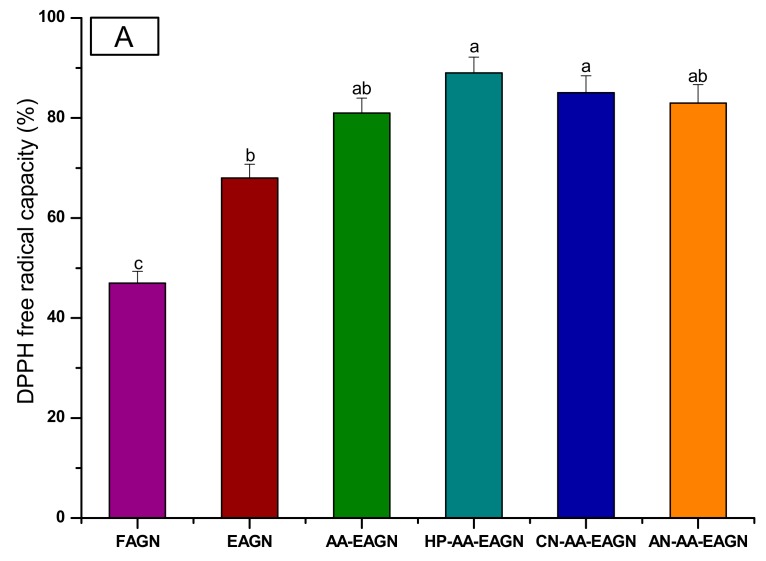

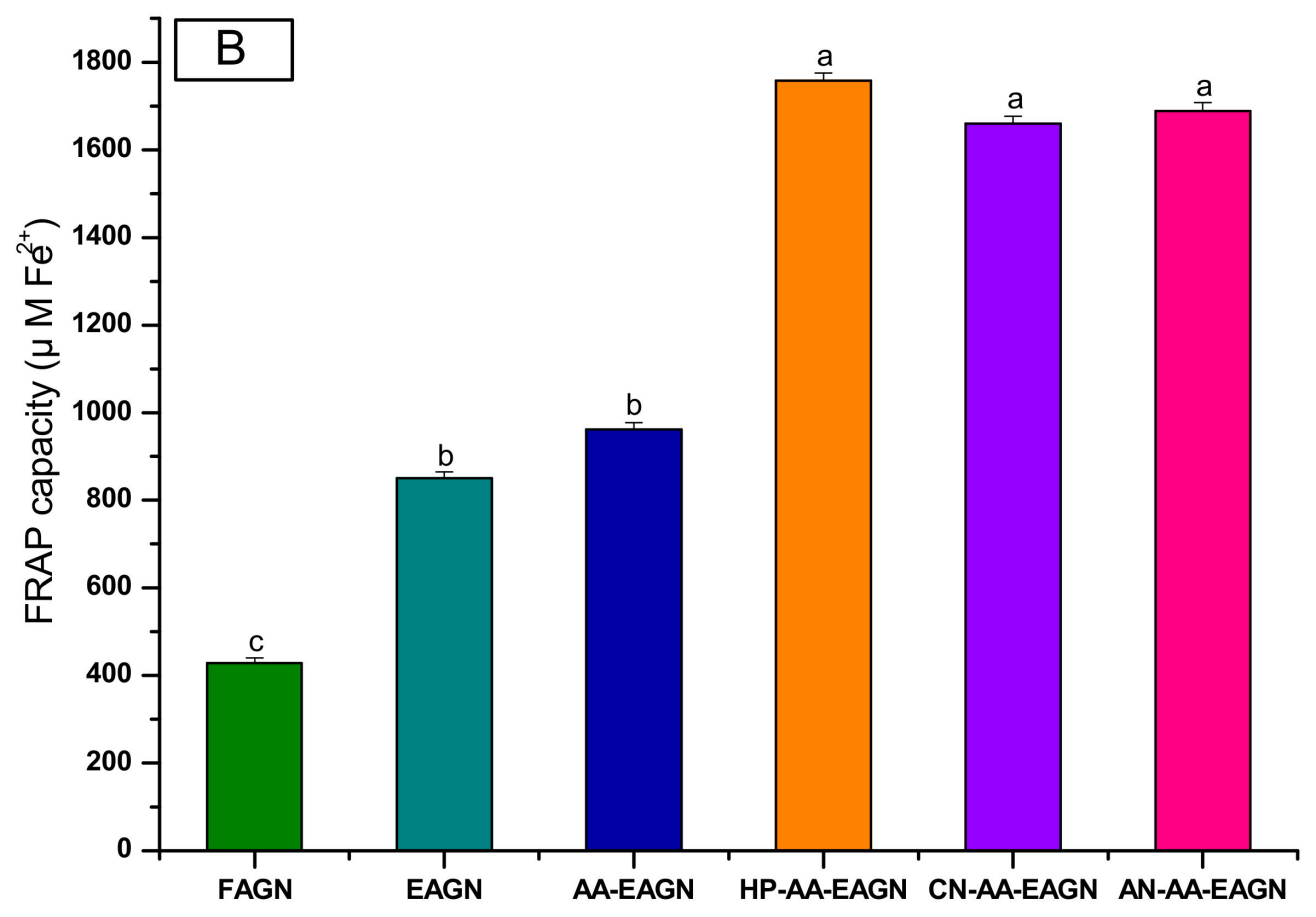
The antioxidant capacity of the AGNC. DPPH (**A**) and FRAP (**B**). Values marked by different letters in each column are significantly different in the *t*-test (*p* < 0.05).

Table 5 shows that the total phenolic (2832 mg/100 g) and total flavonoid (418 mg/100 g) contents were higher in the formulation of HP-AA- EAGN compared to FAGN (1421 mg/100 g, 119.5 mg/100 g, respectively). In the same way, the decursin (148.38 mg/100 g) and decursinol angelate (123.96 mg/100 g) was significantly higher in HP-AA-EAGN compared to FAGN (67.28 mg/100 g, 48.56 mg/100 g, respectively) (Figure 6). Following the phenolic and flavonoid content of the AGNC formulation, the antioxidant capacity was increased in the formulation of HP-AA-EAGN compared to the control measured by the DPPH and FRAP methods (Figure 7).

The high level of compression and shear forces imposed by HME facilitated the extraction of phenolic compounds [50]. Hu et al. [51] stated that the increased extraction of phenolic compounds is due to the strong shear force of extrusion, which partially breaks down the ester bonds and conjugated moieties between phenolics and the cell walls of the complex. The physical crosslinking process of the HME de-structured the fiber matrix and consequently caused phenolic compounds to be released into the solution [52].

HME increases the reactive surface area of compounds and de-structures the fiber matrix, thereby causing enhanced D and DA to be released into the solution (Figure 5), thus contributing to an enhancement of the antioxidant capacity (Figure 7). Moreover, Hagi and Hatami [53] showed that higher levels of flavonoid were produced with the use of an acid-mediated solution derived from vegetables and medicinal plants. The most likely explanation for the enhanced active-compound extraction from the extrudate sample is the disruption of the cell wall structure by the HME, which enables the easier extraction/release of phenolic compounds from samples, thus increasing the total number of phenolic compounds [21]. It is explained that due to hydrophilic characteristics and sol–gel behavior of the HPMC polymer, extraction was facilitated [54].

The increased antioxidant properties after processing might be due to the breakdown of the cellular constituents and the membranes of the materials. The formation of a variety of intermediate byproducts during processing might contribute to improved antioxidant properties [55]. The denaturation of proteins during extrusion leads to open loose structures. These structures promote tannin–protein interaction and form tannin–protein complexes that enhance antioxidant capacity [56]. White et al. [57] observed that antioxidant activity is increased in extrudate products with an elevated barrel temperature. Brennan et al. [58] reported that extrusion may improve the bioavailability of bioactive compounds by forming complexes with proteins, thus yielding an increased level of antioxidant activity in the human body. Studies have reported that the trends of antioxidant activity are generally similar to those of phenolic content [1,59]. This has supported the changes in phenolic compounds and antioxidant activity in this study.

Hu et al. [51] have demonstrated the optimal extraction efficiency of the phenolic compounds from black rice at a temperature range between 110 °C and 120 °C. Temperatures above 120 °C resulted in the degradation of the phenolic compounds due to changes in the molecular structure of the compounds [60].

## 4. Conclusions

In the current study, a hypromellose phthalate biopolymer exhibited the best performance in terms of enhancing the extraction of the phenolic compounds and flavonoids of extrudate *Angelica gigas* Nakai, compared to methods without polymer mixes. It was observed that hot melt extrusion helps to produce nanosized particles, increase solubility, reduce the glass transition energy and temperature, and produce new functional groups. The use of acetic acid as a plasticizer efficiently reduced the glass transition temperature and accelerated amorphization. In brief, hydroxypropyl methylcellulose biopolymer can be used with an acetic acid solution to enhance the functionality of nutraceutical compounds from the *Angelica gigas* Nakai.

## Figures and Tables

**Table 1:** Grade and chemical attributes of the HPMC biopolymer.

Polymer Grade	Chemical Name	Compositions	Generic Name	Molecular Weight	Gelling Temp. (°C)	Bulk Density (g/mL)	Viscosity (m Pa.s) at 20 (°C)	Functional Group
HP55	Cellulose, 2-hydroxypropyl methyl ether phthalic acid ester	HPMC + Glacial acetic acid + Sodium cetate + Phthalic anhydride + Potassium chlorate	Hypro-mellose phthalate	20,000–100,000	40–90	0.31–0.42	32–48	Ether and phthalic acid ester
CN40H	Cellulose, 2-hydroxypropyl methyl ether	High Viscosity HPMC + HCl + H_2_O	Hypro-mellose	10,000–1,000,000	40–90	0.30–0.52	4000	Ether
AN6	Cellulose, 2-hydroxypropyl methyl ether	Low Viscosity HPMC + HCl + H_2_O	Hypro-mellose	10,000–1,000,000	40–90	0.30–0.52	6	Ether

Source: www.lotte-cellulose.com. Biopolymers used in this study was donated by the Lotte Chemical Co.

**Table 2:** Composition of *Angelica gigas* Nakai (AGN)–biopolymer–plasticizer of the AGN–biopolymer–plasticizer composite (AGNC) formulation.

Materials	Mixing Ratio (*w*/*w*)	HME Condition	HME Temp. (°C)	Formulation
Fresh AGN powder	100	Non extrusion	--	FAGN
AGN powder	100	Extrusion	80/100/120/80	EAGN
AGN + Acetic acid (AA)	100	Extrusion	80/100/120/80	AA-EAGN
AGN + AA + HP55	95-5	Extrusion	80/100/120/80	HP-AA-EAGN
AGN + AA + CN40H	95-5	Extrusion	80/100/120/80	CN-AA-EAGN
AGN + AA+AN6	95-5	Extrusion	80/100/120/80	AN-AA-EAGN

**Table 3:** Particle size distribution of the AGNC formulation.

Formulations	Particle Size (nm)
FAGN	1499 ± 5.4 a
EAGN	478 ± 3.1 b
AA-EAGN	448 ± 3.3 b
HP-AA-EAGN	341 ± 3.4 c
CN-AA-EAGN	354 ± 2.7 c
AN-AA-EAGN	323 ± 2.1 c

FAGN: fresh non-extrudate AGN; EAGN: extrudate AGN; AA-EAGN: acetic acid (0.1 M) mediated EAGN; HP/AN/CN-AA-EAGN: HPMC (5% *w*/*w*) mediated AA-EAGN. Values marked by different letters in each column were significantly different during the *t*-test (*p* < 0.05).

**Table 4:** Water solubility of the AGNC formulation.

Formulations	WAI	WS (%)	SP
FAGN	4.41 ± 0.50 a	29.69 ± 0.94 d	9.52 ± 1.31 a
EAGN	3.27 ± 0.41 b	42.54 ± 1.24 c	5.31 ± 1.28 b
AA-EAGN	3.75 ± 0.42 b	51.35 ± 1.49 b	5.65 ± 1.42 b
HP-AA-EAGN	2.63 ± 0.93 c	65.21 ± 1.28 a	4.61 ± 0.88 c
CN-AA-EAGN	2.54 ± 0.86 c	59.34 ± 2.13 a	4.15 ± 0.23 c
AN-AA-EAGN	2.59 ± 0.43 c	61.46 ± 1.91 a	4.36 ± 0.71 c

WAI: water absorption index; WS: water solubility; SP: swelling power. Values marked by different letters in each column are significantly different in the *t*-test (*p* < 0.05).

**Table 5:** Phenolic content and total flavonoid content of the AGNC formulation.

AGNC Formulations	Total Phenol (mg/100 g)	Total Flavonoid (mg/100 g)
FAGN	1421.0 ± 88.7 c	119.5 ± 1.2 d
EAGN	1649.2 ± 59.2 b	138.1 ± 5.4 c
AA-EAGN	1684.7 ± 48.3 b	179.2 ± 1.4 b
HP-AA-EAGN	2832 ± 62.6 a	418.5 ± 22.2 a
CN-AA-EAGN	2725 ± 46.24 a	298 ± 24.14 a
AN-AA-EAGN	2788 ± 55.94 a	306 ± 13.74 a

Values marked by different letters in each column are significantly different in the *t*-test (*p* < 0.05).
